# Robust CA-GO-TiO_2_/PTFE Photocatalytic Membranes for the Degradation of the Azithromycin Formulation from Wastewaters

**DOI:** 10.3390/polym16101368

**Published:** 2024-05-10

**Authors:** Veronica Satulu, Andreea Madalina Pandele, Giovanina-Iuliana Ionica, Liliana Bobirică, Anca Florina Bonciu, Alexandra Scarlatescu, Constantin Bobirică, Cristina Orbeci, Stefan Ioan Voicu, Bogdana Mitu, Gheorghe Dinescu

**Affiliations:** 1 National Institute for Laser, Plasma and Radiation Physics, 409 Atomistilor Str., 077125 Magurele, Romania; veronica.satulu@inflpr.ro (V.S.); anca.bonciu@inflpr.ro (A.F.B.); alexandra.scarlatescu@inflpr.ro (A.S.); dinescug@infim.ro (G.D.); 2 Faculty of Chemical Engineering and Biotechnologies, National University of Science and Technology Politehnica Bucharest, Polizu 1-7, 011061 Bucharest, Romania; madalina.pandele@upb.ro (A.M.P.); giovanina.lupu@upb.ro (G.-I.I.); liliana.bobirica@upb.ro (L.B.); constantin.bobirica@upb.ro (C.B.); cristina.orbeci@upb.ro (C.O.); 3 Advanced Polymers Materials, National University of Science and Technology Politehnica Bucharest, 011061 Bucharest, Romania; 4 Faculty of Physics, University of Bucharest, 077125 Magurele, Romania

**Keywords:** cellulose acetate with TiO_2_-decorated graphene oxide membranes, polytetrafluorethylene thin films, wastewater treatment, photocatalysis, azithromycin formulation

## Abstract

We have developed an innovative thin-film nanocomposite membrane that contains cellulose acetate (CA) with small amounts of TiO_2_-decorated graphene oxide (GO) (ranging from 0.5 wt.% to 2 wt.%) sandwiched between two polytetrafluoroethylene (PTFE)-like thin films. The PTFE-like films succeeded in maintaining the bulk porosity of the support while increasing the thermal and chemical robustness of the membrane and boosting the catalytic activity of TiO_2_ nanoparticles. The membranes exhibited a specific chemical composition and bonding, with predominant carbon–oxygen bonds from CA and GO in the bulk, and carbon–fluorine bonds on their PTFE-like coated sides. We have also tested the membranes’ photocatalytic activities on azithromycin-containing wastewaters, demonstrating excellent efficiency with more than 80% degradation for 2 wt.% TiO_2_-decorated GO in the CA-GO-TiO_2_/PTFE-like membranes. The degradation of the azithromycin formulation occurs in two steps, with reaction rates being correlated to the amount of GO-TiO_2_ in the membranes.

## 1. Introduction

Water scarcity is a worldwide concern that severely impacts the health and wellbeing of all life on Earth [1,2]. With the rapid growth of industry and urbanization, there has been a decrease in the number of forests, coupled with an increase in population, which has led to a rise in pollution at a global level. Concerns arise from the rapid increase in wastewater generation and the escalating need for clean water, which indicate an imminent international crisis related to water availability [3]. To address this, policymakers have proposed Sustainable Development Goals (SDGs), and much effort is being placed into evaluating and monitoring global initiatives regarding wastewater treatment [4]. Polluting organic compounds, especially those of a synthetic origin, that are widely used in industrial applications have become a recent environmental problem [5]. In particular, pharmaceutical compounds are a class of organic substances that have become progressively important in modern human science because of their ability to cure a significant number of diseases and improve the quality of human medication [6]. As a result of their steady growth in usage throughout time, they have become one of the leading environmental contaminants entering the water cycle [7]. In recent years, due to the COVID-19 pandemic, which has led to an upsurge in the consumption of some specific drugs, several compounds have been found in wastewater, originating from the pharmaceutical industry, hospitals, and even urban water sources [8]. During the coronavirus pandemic, one of the most commonly used antibiotics was azithromycin, which was available in tablet form or as a powder in suspension [9]. Azithromycin is a broad-spectrum bacteriostatic antibiotic used to treat respiratory tract conditions as well as skin and soft tissue infections caused by Gram-positive pathogens [10]. Due to its intensive use, this antibiotic now appears in municipal wastewater, which requires proper treatment to reduce residual compounds [9]. The conventional treatment technologies for removing large molecules, such as azithromycin, have not yielded promising results [11,12,13,14]. However, the use of polymeric membranes for separation has shown significant potential in removing organic pollutants from water and wastewater during purification processes [15,16,17,18]. Retention techniques based on membrane processes have been found to provide better antibiotic adsorption efficiency, up to 80–85%, compared to conventional methods [12,13,14,19]. Advanced oxidation processes (AOPs), on the other hand, are designed to degrade toxic organic compounds by mineralizing them and transforming them into intermediate products [19]. Given the projected increase in water consumption and drug use (including antibiotics, antiviral drugs, antidepressants, analgesic hormones, and others), it is imperative to eliminate or degrade contaminants from wastewater [20]. To this end, combining separation via a membrane with advanced oxidation processes is a promising approach [21]. Semiconductor materials, such as TiO_2_, ZnO, and WO_3_, are extensively used to eliminate organic pollutants from wastewater by photocatalysis [22]. Among these, TiO_2_ is a widely used semiconductor due to its good photocatalytic activity, high redox potential, and chemical stability. However, TiO_2_ has certain limitations, including charge carrier recombination and poor responsiveness to visible light. These challenges are being addressed by scientists who are exploring various approaches to overcome them. For instance, some researchers are investigating the use of doping techniques to enhance the photocatalytic activity of TiO_2_, while others are exploring the use of co-catalysts to improve its responsiveness to visible light. The use of TiO_2_ catalysts decorated onto carbon-based materials represents another promising strategy to modulate their response to the visible light spectrum [23]. Graphene oxide (GO) has received considerable attention due to its significant properties, such as a large surface area, good conductivity, ability to adsorb organic impurities, and extended visible light absorption range [24]. Composite membranes frequently use GO as a filler due to its outstanding mechanical and thermal properties, and due to its benefits in terms of water flow and separative performance [25]. There are various methods for obtaining metal oxide-decorated graphene oxide [26], with the most common being solution mixing [27], atomic layer deposition [28], sol–gel or self-assembly [29], solvothermal [30], and electrochemical deposition [31]. A simple method was reported for producing TiO_2_-decorated carbon nanotubes using a sonochemical approach [32]. The method involves the decoration of carbon nanotubes with TiO_2_ particles by sonication in a polymer solution (polysulfone in N,N′-dimethylformamide), followed by the synthesis of a membrane through phase inversion. A composite membrane of polysulfone-TiO_2_-decorated carbon nanotubes was produced and utilized for the photocatalytic degradation of ampicillin and erythromycin [32].

Furthermore, the field of membrane science is increasingly embracing circular economy principles by replacing synthetic polymers with natural ones or their derivatives [33]. Among these, cellulose and its derivatives are the most researched and preferred for water treatment due to their high natural abundance [34]. Cellulose acetate (CA) is commonly used for membrane synthesis due to its easy and cost-effective production, as well as for its high solubility in polar organic solvents. Despite its advantages, the use of this material is limited by issues related to fouling and reduced chemical resistance [35]. Several studies have recently been conducted to improve membrane performance, including increasing water flux and enhancing the rejection of targeted materials while minimizing fouling and maintaining physical and chemical stability [36]. One route to achieve these benefits is by synthesizing composite membranes [37,38].

Thin-film composite (TFC) membranes have been proven to be highly effective in various wastewater treatment processes. Unlike traditional polymeric membranes, TFC membranes have a thin top coating layer that allows for greater efficiency [39]. Fluoropolymer materials are highly attractive for membrane coating due to their excellent chemical resistance, thermal stability, and oxidative stability [36]. Among the various fluoropolymers, polytetrafluoroethylene (PTFE) stands out for its desirable properties, such as a low dielectric constant, high chemical resistance, good electrical stability, low coefficient of friction, excellent mechanical properties, and remarkable stability across a wide range of temperatures and humidities. Furthermore, PTFE showcases remarkable piezoelectric properties in combination with TiO_2_ nanospheres [40], making it a promising material for energy harvesting and environmental remediation. Due to its exceptional chemical resistance, as well as thermal and oxidative stability, PTFE is considered one of the most promising materials for the synthesis of thin-film composite membranes [41]. There are various ways to create thin films using solid targets of fluorine-containing materials. Some of these methods include electron beam irradiation [42], thermal chemical vapor deposition [43], and vacuum evaporation [44]. In addition, a useful technique is radiofrequency magnetron sputtering, in which atoms, oligomers, and volatile fragments removed from a PTFE target are released into the plasma volume and condense as a thin film with a similar composition as that of a target during the polymerization process at the surface [45,46]. Compared to previously mentioned methods [37,38,39], this last deposition technique offers advantages such as good process control, better uniformity of the thin films, and larger deposition areas, as emphasized in [47]. The combination of the piezoelectric properties of PTFE material with the catalytic activity of TiO_2_ nanoparticles creates a piezo-photocatalytic composite system that significantly enhances TiO_2_’s catalytic performance [40]. When the composite system is exposed to light, electrical charges are created at interfaces that can drive catalytic reactions. This advanced technology has the potential to revolutionize various sectors such as solar power, sensors, and wastewater treatment [40]. Consequently, the development and fine-tunning of photocatalytic membranes are essential for addressing environmental challenges and advancing sustainable technologies.

The main objective of this study was to develop a thin-film composite membrane based on cellulose acetate, with high-temperature performance and UV resistance, that is suitable for the photocatalytic degradation of antibiotics. We propose a commonly used method that combines the advantages of sonochemical mixing with phase inversion for the decoration of GO with TiO_2_ nanoparticles and their inclusion into cellulose acetate membranes. In addition, we use chemically resistant PTFE-like thin films deposited on the membranes’ sides to enhance the robustness of the porous support. We show that the composite system based on PTFE-like material and TiO_2_ nanoparticles exhibits unique properties that improve the catalytic performance of TiO_2_. Upon exposure to light, the piezoelectric effect of the PTFE-like material is triggered, generating an electrical charge that propels catalytic reactions. This synergy between PTFE-like material and TiO_2_ has the potential to significantly enhance the efficiency of catalytic systems via wastewater treatment through the photodegradation of the azithromycin formulation, as exemplified in the following sections.

## 2. Materials and Methods

### 2.1. Materials and Chemical Reagents

Cellulose acetate, with a molecular weight of 34,000 Da; titanium dioxide (TiO_2_, nanopowder, 20 nm size, Degussa P25: 80% anatase 20% rutile); and graphene oxide (GO) were purchased from Sigma-Aldrich (St. Louis, MO, USA). N,N′-dimethylformamide (DMF) and ethanol with analytical purity were purchased from Merck KGaA (Darmstadt, Germany) and used without further purification processes. For machining magnetron targets according to the technical specifications of the sputtering gun (diameter: 25.4 mm, thickness: 3.18 mm), we used a PTFE rod (Goodfellow, FP307980, Cambridge, UK). Before being mounted on the magnetron gun, the PTFE targets machined correspondingly were cleaned via ultrasonication in laboratory-grade ethanol, and they were dried with compressed N_2_ (4.6 purity). For this study, an Azitrox formulation, a powder for oral suspension with a concentration of 200 mg/5 mL of the antibiotic azithromycin, was purchased from a pharmacy (producer Zentiva S.A., Bucharest, Romania). It consists of azithromycin dihydrate, which is the the active component, and several organic and inorganic excipients, including sugar, trisodium phosphate anhydrous, hydroxypropyl cellulose, xanthan gum, and banana flavoring. In the rest of this paper, the abbreviation AZT will be used to refer to this antibiotic formulation. A solution with a 30% concentration of H_2_O_2_ (hydrogen peroxide) was purchased from Sigma-Aldrich and used as a source of hydroxyl radical species during catalysis. Once the working electrochemical solution was prepared, we adjusted its pH to the default value by adding an appropriate amount of the H_2_O_2_ solution.

### 2.2. Sample Preparation

#### 2.2.1. Sonochemical and Phase Inversion Methods for the Synthesis of TiO_2_-Decorated GO and Composite Membranes

**Figure 1:**
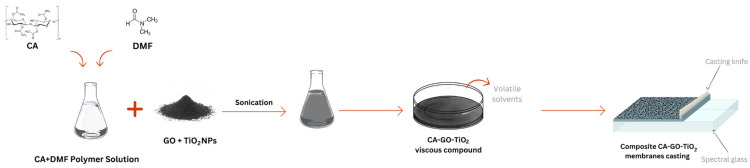
Illustration of the steps involved in the preparation of the CA-GO-TiO_2_ membranes.

To prepare the polymer solution, cellulose acetate was dissolved in N,N′-dimethylformamide (DMF) at a concentration of 12 wt.% The solution was magnetically stirred for 24 h at room temperature to ensure complete homogenization. For the preparation of the composite membranes, graphene oxide and TiO_2_ nanoparticles were added to the solution. As the distribution of TiO_2_ is expected to be dependent on the concentration of graphene oxide, three different concentrations of graphene oxide were used for the preparation of composite membranes—0.5%, 1%, and 2 wt.%—relative to the polymer mass in the solution. The process of decorating graphene oxide with titanium dioxide and dispersing the decorated nanofiller in a polymer solution was carried out in one step using sonication for 30 min while keeping the temperature below 5 °C by cooling the admixture in an ice bath. After 30 min of sonication, a homogeneous, slightly viscous, black mass was obtained. To form the membranes, a phase immersion method was used. For that, the polymer solution was deposited onto a spectral glass with a fixed blade thickness of 250 μm. The spectral glass was then immersed in a coagulation bath containing 200 mL of distilled water until the membranes were separated from the support’s surface. After synthesis, the obtained membranes were washed with deionized water and ethanol [32]. Upon phase inversion synthesis of the membranes, they showed asymmetric pores, presenting small pore openings on one side and bigger pore openings on the opposite side. For the experiments, where photocatalytic degradation was foreseen, one requirement was to efficiently expose the membrane to UV light. Therefore, we considered the active side of the membrane to be the one with bigger pores. Figure 1 presents a schematic of the main steps involved in the initial membrane preparation process.

#### 2.2.2. Plasma Deposition of PTFE-like Films

**Figure 2:**
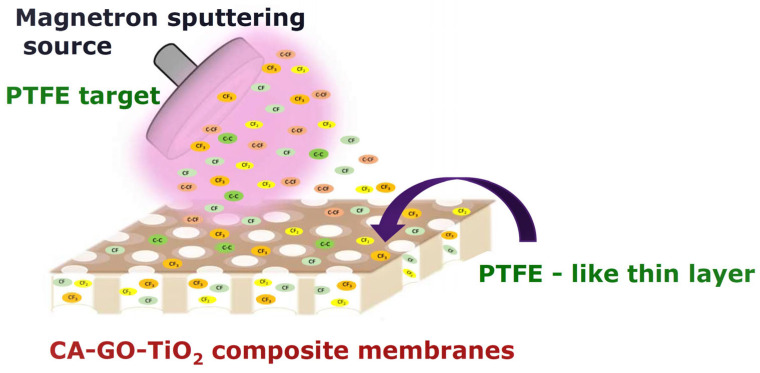
Schematic illustration of the deposition process of PTFE-like thin films via magnetron sputtering for the creation of composite membranes with superior chemical robustness; the different colored ovals in the diagram representing the film describe the incorporation of different species and radicals, suchas C-C, CF, C-CF, CF_2_, and CF_3_, in the deposited material.

The deposition of PTFE-like films was carried out in a stainless-steel vacuum chamber with a spherical shape. The chamber was equipped with a 1″ magnetron sputtering source (Kurt J. Lesker) and described in detail [48]. The magnetron source was mounted on a flange tilted at a 45° angle with respect to the normal of the substrate surface at a distance of 6 cm, which was determined from the central axis of the magnetron gun to the central axis of the rotating substrate holder. The substrate was not heated during the deposition process, and a slight increase in the temperature during the deposition process was possible due to plasma exposure. The deposition process involved operating the discharge with 80 W RF power (13.56 MHz) in argon (purity 99.9999%), at a flow rate of 100 sccm, and under continuous pumping. This established a working pressure of 1.4 × 10^−3^ mbar. The magnetron source was technically adjusted by adding a chimney in front of the magnetron ring to prevent poisoning the target. Based on the previous measurements using the contact profiler technique, it was determined that the deposition rate for the experimental conditions mentioned above should be 4 nm/min. To obtain thin-film composite membranes, the sputtering time was set to 50 min, leading to 200 nm of thickness of PTFE-like layers on the side devoted to face the UV radiation (called the ‘active side’ hereafter). Five minutes of deposition was applied on the opposite side of the membrane to achieve hydrophobization and for the process to favor the transfer between the oxygen species of TiO_2_ and the fluorine species from the sputtering PTFE plasma [49,50,51]. This deposition time corresponds to a 20 nm thick film. The process of magnetron sputtering deposition of PTFE-like material onto the membranes is illustrated in Figure 2.

### 2.3. Investigation of the Properties of the Thin-Film Composite Membranes

High-Resolution Field Emission Scanning Electron Microscopy (HR-FE-SEM) was used to evaluate the morphology of the initial support of the membranes and the modification of pores after the deposition process. The measurements were carried out on an Apreo S LoVac apparatus from Thermo Fisher Scientific (Waltham, MA, USA), which operated at an acceleration voltage of 10 kV. Prior to the analysis, a 10 nm thick layer of gold was applied to the surface of the composite membranes to prevent charge accumulation during the examination process.

The apparent pore diameter of the membranes was evaluated using Adobe Photoshop software (version 25.51, Adobe Inc., San Jose, CA, USA, 2024) to enhance pore boundary demarcation, and ImageJ software (version 1.54g, NIH Image, Washington, DC, USA) was used for area fraction measurements. To obtain an accurate statistical analysis, we determined the values using the average apparent pore diameters based on the measurements of 50 independent pores on each sample, and we reported the obtained standard deviation for each experimental condition.

The evaluation of the total porosity of each membrane was performed using the X-ray microtomography method (μCT) with a Bruker SkyScan 1272 microCT machine (Bruker microCT, Kontich, Belgium). Rectangular specimens were cut from the middle of each membrane, with dimensions of approximately 5 mm in length and 3 mm in width. The images were captured with a resolution of 2 μm per pixel and a rotation step of 0.2°, with an average of 10 frames per capture. The obtained data were computed to generate a 3D image of the membrane and its characteristics were analyzed using CTAn software (Kyscan 1272) [52].

The surface chemical composition of the initial CA-GO-TiO_2_ support membranes and CA-GO-TiO_2_/PTFE composite membranes was analyzed using X-ray Photoelectron Spectroscopy (XPS), in an as-synthesized state. XPS analysis was conducted using a K-Alpha Thermo Scientific (ESCALAB™ XI+, East Grinstead, UK) spectrometer equipped with a 180° double-focusing hemispherical analyzer. The peak positions were calibrated with respect to the adventitious C1s peak at 284.8 eV, as indicated by Avantage data software (Thermo Avantage v5 9921, East Grinstead, UK). Surface elemental composition was determined by recording survey spectra at a pass energy of 50 eV. The elemental bonding states of the obtained CA-GO-TiO_2_/PTFE composite membranes were evaluated by measuring high-resolution spectra for the binding energy regions of C1s, O1s, and F1s at a pass energy of 20 eV [53]. The acquisition and processing of spectra were performed using the above-mentioned advanced Avantage data software.

The chemical structure of the CA-GO-TiO_2_ support membranes and CA-GO-TiO_2_/PTFE composite membranes were analyzed using Fourier transform infrared spectroscopy (FTIR) with Bruker Vertex 70 equipment (Bruker, Billerica, MA, USA) and a diamond ATR device. Vibrational spectroscopy analysis was conducted within the range of 600–4000 cm^−1^ by recording the spectra as an average of 32 successive measurements, with a resolution of 4 cm^−1^, leading to an increase in signal-to-noise ratio. Further spectral processing including ATR correction, baseline correction, and Savitzky–Golay filtering was applied to additionally analyze the spectral features of the material. The specific spectral bands associated with atmospheric CO_2_ and water vapor were removed via automatic software processing [54].

The stability of the membranes was evaluated based on the results obtained in the photocatalytic degradation tests of the Azitrox formulation. Thermal stability was investigated using thermal gravimetric analysis (TGA). TGA was performed by utilizing TA Instruments Q500 equipment (Bellingham, WA, USA) (under a nitrogen atmosphere with a heating rate of 10 °C/min from room temperature to 800 °C [54].

**Figure 3:**
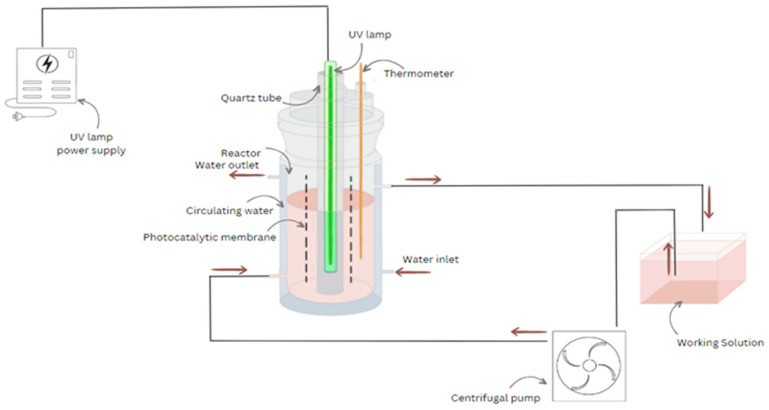
Schematic illustration of the reactor for photocatalytic oxidation with continuous recirculation.

Photocatalytic oxidation assessments were conducted in the set-up schematically described in Figure 3, for which the working parameters are specified in Table 1. Hydrogen peroxide was added to the reaction mixture, and UV radiation was applied by activating the UV lamp of the photocatalytic reactor. Each photocatalytic membrane was twisted around the UV lamp to ensure proper light exposure and was then evaluated in the photocatalytic reactor. The working solution was continuously recirculated at room temperature using an external centrifugal pump, and a recirculation vessel was used to facilitate the process.

The effectiveness of photocatalytic degradation was evaluated by monitoring the changes in the organic content of the solution over irradiation time. Liquid samples comprising 10 mL were extracted from the reactor at specified times to examine the efficiency of degradation. To analyze organic content, chemical oxygen demand (COD) was measured using the APHA 5220 D standard method) [55] (closed reflux, colorimetric method. In short, the aqueous solution samples are digested for two hours at 150 °C in a strong oxidizing medium of potassium bichromate and sulfuric acid. During this time, the dichromate ions oxidize the organic material (expressed as COD) in the sample, and chromium passes from the hexavalent form (Cr^6+^) to the trivalent form (Cr^3+^). Next, the samples are cooled and measured at a wavelength of 600 nm, where the chromic ion absorbs strongly, and the dichromate ion practically does not absorb at all. According to the mentioned standard, the calibration curves were obtained by using the standard potassium hydrogen phthalate solution with COD equivalents in the range of 20 to 900 mg/L. The LT 200 thermostat and DR 3800 spectrophotometer from Hach Lange GmbH, Weinheim, Germany, were utilized to ensure an accurate and successful COD analysis.

## 3. Results

### 3.1. The Morphological and Structural Properties of the CA-GO-TiO_2_ and CA-GO-TiO_2_/PTFE Membranes

#### 3.1.1. Surface Morphology

**Figure 4:**
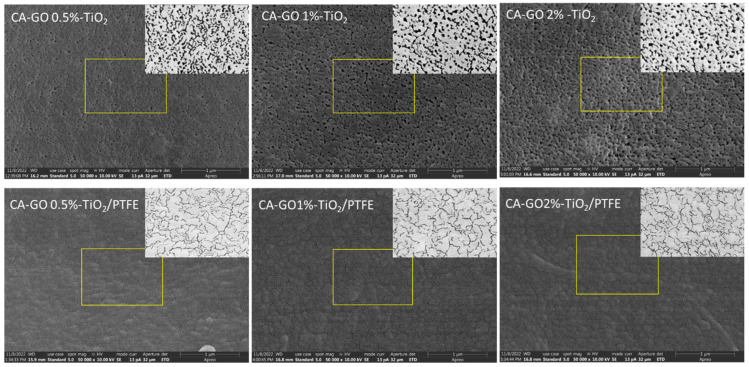
SEM images (magnification 50,000×) of the initial CA-GO-TiO_2_ membranes’ surfaces with various TiO_2_-decorated GO concentrations and of TFC composite membranes coated with a 200 nm PTFE-like layer. The yellow boxes identify the processed regions of the SEM images presented in the corner of each image in order to better evidence the sample porosity before and after the PTFE-like coating.

Figure 4 shows Scanning Electron Microscopy (SEM) images of the active side of the membranes’ surfaces obtained for various GO-TiO_2_ concentrations. The SEM micrographs indicate that all investigated initial samples have similar morphologies, with nanometric size quasi-circular pores being open on the surface.

After the deposition of the PTFE-like thin film via magnetron sputtering onto the membranes’ surfaces, the SEM images reveal a significant morphological transformation from circular to channel-like surface openings. This change can be attributed to several factors related to the growth dynamics of the PTFE-like material on the CA-GO-TiO_2_ membrane with non-homogeneous surface distribution of the initial pores. Given the high surface area and the porous nature of the substrate, species and radicals originating from the sputtering of the PTFE target begin to settle on the flat part of the surface and within the pores of the membrane. Due to the surface energy differences and the chemical nature of the substrate, PTFE prefers to nucleate on specific sites that offer the lowest energy barrier for adsorption. At this stage, isolated PTFE islands form on the surface. As deposition continues, these PTFE islands grow and start to coalesce, merging to form a continuous film. This growth leads to flat regions (those without pores) being coated in thin film and to a partial covering of the pores, effectively reducing their visibility and size in SEM images. Moreover, as the islands merge, they may span across the openings of pores, leading to the formation of narrow channel-like structures atop the membrane. These channels are formed due to the lack of bridging of PTFE over close consecutive pores, creating a network of tunnel-like features on the membranes’ surfaces. Film cracking above the covered pores caused by the mismatch in the properties of the two materials may also contribute to these features.

Aiming to evaluate the surface porosity, each SEM image was processed to demarcate the pores and channels boundaries, as shown in the insets from Figure 4. Table 2 presents the percentage of the surface area of pores openings (seen in black in the inset images) computed from the insets. According to the values in Table 2, the surface porosity of initial CA-GO-TiO_2_ membranes is in the range of 20–25%, decreasing slightly as the GO-TiO_2_ concentration increases. After coating with the PTFE-like material, the surface porosity was reduced by about 40%, and was in the range of 12–15%.

#### 3.1.2. Structural Properties

**Figure 5:**
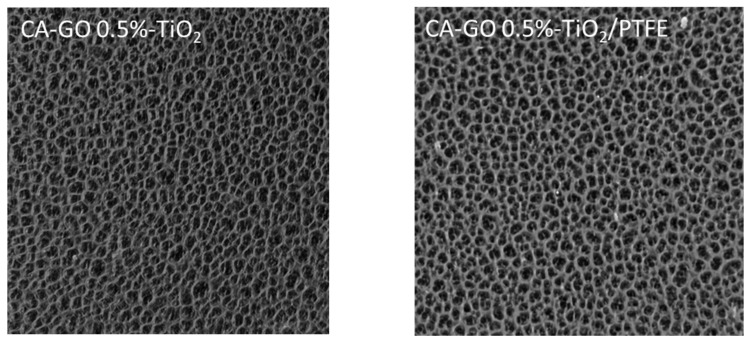
Cross-sections through 3D reconstruction of the microtomographies of the initial CA-GO0.5%-TiO_2_ and coated CA-GO0.5%-TiO_2_/PTFE; lateral dimension in the reconstructed image is 250 μm on both sides.

X-ray microtomography (μCT) was performed to obtain information about the internal structure and porosity of both the CA-GO-TiO_2_ and CA-GO-TiO_2_/PTFE membranes. Figure 5 depicts, as examples, cross-sections across 3D reconstruction images of CA-GO0.5%-TiO_2_ and CA-GO0.5%-TiO_2_/PTFE membranes. Visually, both samples show an internal porous structure consisting mostly of opened and interconnected pores, without peculiar distinguishable features. This similarity is explained by the position of the cross-section at a large distance from the membrane’s external sides, where the very thin PTFE-like film was applied and where the penetration of the PTFE coating species was impeded.

Still, via a detailed statistical analysis of the μCT images, it is possible to determine average characteristics, like the thicknesses of the structural components (i.e., the average width of the pore walls), the specific surface area (surface area-to-volume ratio), total porosity, and closed porosity, for each sample. The results of these calculations are presented in Table 3. Noticeably, the corresponding quantities for each sample vary without clear monotonic trends in respect to the amount of incorporated GO-TiO_2_ or the presence of the PTFE-like layer. The spreading of the results reflects the inherent variations in samples obtained with the phase inversion method (formation of pores is a statistical process) combined with the resolution limitations of X-ray microtomography.

From the average values presented in Table 3, pertinent conclusions can be drawn on the effect of the PTFE-like layer. Compared with the average values of the initial CA-GO-TiO_2_ membranes, the coated membranes present an increased structure thickness, increased closed porosity, decreased surface area, and decreased total porosity. These results corroborate well with the peculiarities of the PTFE-like coating process, which modifies the upper layers of the membranes, deeply penetrating and partially closing the pores accessible from the surface.

### 3.2. Chemical Characterization of the CA-GO-TiO_2_ and CA-GO-TiO_2_/PTFE Membranes

**Figure 6:**
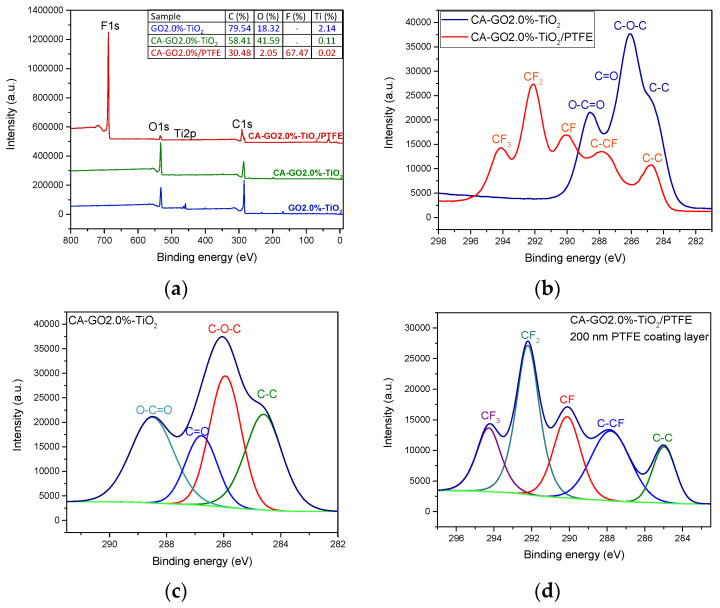
Comparative XPS spectra for the initial and PTFE-like coated CA-GO-TiO_2_ membranes: (**a**) survey spectra and elemental concentration evidencing the modification of surface chemistry; (**b**) binding energy region and bonding identification of C1s; (**c**) deconvolution of the initial CA-GO2%-TiO_2_ membrane with the labeling of the specific peaks; (**d**) deconvolution of the CA-GO2%-TiO_2_/PTFE with the peak labels that evidence the formation of a PTFE-like coating on the membrane’s surface.

Information on chemical bonding and the bonds’ dependencies on the experimental conditions used during membrane synthesis are presented in Figure 6, with this information being obtained from XPS investigations.

Based on the XPS survey spectra in Figure 6a, it is evident that the primary elements in the initial CA-GO-TiO_2_ support membranes are carbon and oxygen. In contrast, the surface of the CA-GO-TiO_2_/PTFE composite membranes contains mainly carbon and fluorine, with only a minimal amount of oxygen as a contaminant element. The Ti element was not observed in the initial membrane surface, so we concluded that its concentration was below the detection limit of the XPS instrument. Nevertheless, to verify the existence of titanium oxide within the composite membranes, we prepared a 1% TiO_2_ suspension in a 2% GO solution and performed XPS investigations on dried suspension samples. The results, which are also presented in Figure 6a, confirm the presence of Ti, corresponding to an atomic concentration of titanium dioxide of 2%, indicating a 0.02% concentration of TiO_2_ in the volume of the composite membrane. This supplementary investigation allowed for a comprehensive assessment of the composite membranes’ chemical composition in terms of the presence of titanium dioxide, a key factor for the photocatalytic efficiency of the membranes, and it confirmed that the XPS detection limit of 0.1% is not sufficient to quantify the TiO_2_ concentration accurately in the samples used in this study.

In Figure 6b, one can compare the high-resolution spectra of the initial support membrane and of the PTFE-like coated one. These spectra confirm the transformation of the membrane surface from one based on C-O bonds to one based on C-F bonds. Furthermore, the advanced processing of the high-resolution spectra was utilized to quantitatively assess the chemical bonding on the surface of the new composite CA-GO-TiO_2_ membranes. Figure 6c displays the high-resolution spectra of C1s for the CA-GO-TiO_2_ composite membranes, including their deconvolution into four components (corresponding to C-C bonds at 284.8 eV, C-O-C bonds at 286.3 eV, C=O bonds at 287.7 eV, and O-C=O bonds at 289 eV), which accounts for both CA and GO components. Comparatively, the surface chemical bonding of the CA-GO-TiO_2_/PTFE-coated membranes was evaluated (Figure 6d) via deconvolution of the C1s high-resolution spectra into five components [46]. These components correspond to the CF_3_ bond at 294 eV with a contribution of 17%, CF_2_ bond at 292 eV with the most significant contribution of 35%, CF bond at 290 eV with a contribution of 20%, and C-CF bond at 288 eV and C-C bond at 285 eV with lower contributions of 16% and 12%, respectively.

**Figure 7:**
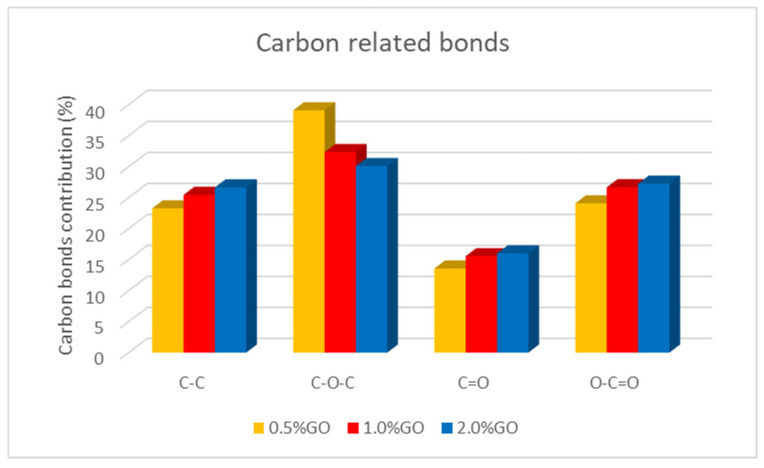
Dependence of the carbon-based bonds as a function of the GO concentration in the CA-GO-TiO_2_ membranes.

We also investigated the influence of the graphene oxide (GO) concentration on the CA-GO-TiO_2_ composite support chemical structure. Figure 7 presents the dependence of the relative contribution of various carbon-related bonds when the GO concentration in the membrane increases. The contribution of C-C bonds increases, along with the contributions of the C=O and O-C=O bonds, indicating an appropriate and homogeneous combination of graphene oxide with cellulose acetate. In contrast, the concentration of C-O-C bonds decreases.

**Figure 8:**
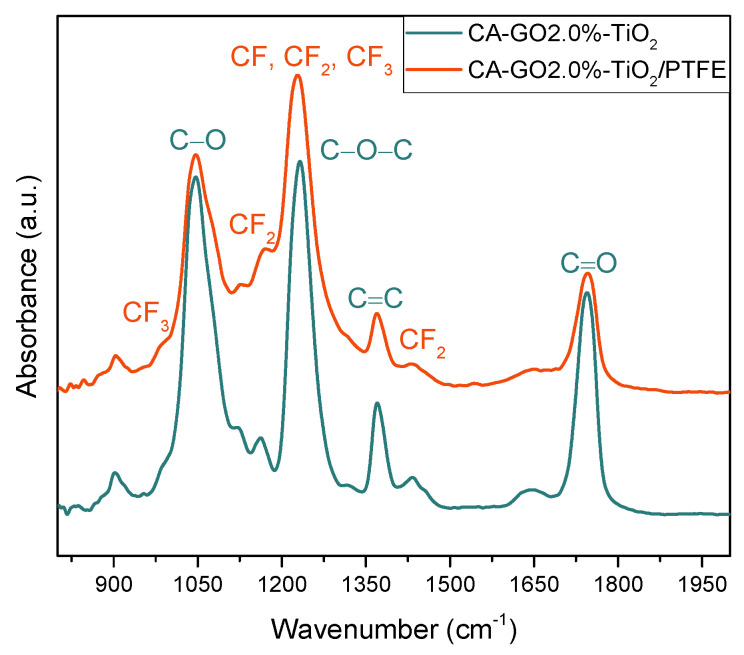
FTIR spectra of the CA-GO 2.0%-TiO_2_ composite membrane (green line) and CA-GO 2.0%-TiO_2_/PTFE thin-film composite membrane (red line).

In addition, the FTIR method was utilized to determine the overall composition of the membranes and corroborates the results obtained from the XPS examination of the membrane surface. Figure 8 presents the FTIR spectra for the composite membranes, namely the CA-GO 2.0 wt.%-TiO_2_ and CA-GO 2.0 wt.%-TiO_2_/PTFE thin-film composite membranes. The cellulose acetate with graphene oxide doped with TiO_2_ (in blue color) typically shows four strong and narrow absorption bands, indicating the main bonds of the compound. These bands are assigned as follows: the 1046 cm^−1^ absorption band is associated with the C-O-C band in the graphene oxide [56] and cellulose backbone, the peak at 1230 cm^−1^ is indicative of the C-O bond’s stretching vibration, while the absorption bands at 1371 cm^−1^ and 1745 cm^−1^ represent the contributions of the C-H bending vibration and C=O stretching in the acetyl group, respectively [57,58,59]. When analyzing thin-film composite membranes, the PTFE-like fingerprint can be identified with the specific absorption bands highlighted in red color. The CF_2_ symmetric and asymmetric stretching bonds are represented by a strong vibration at 1227 cm^−1^ and a small shoulder at 1170 cm^−1^. Additionally, CF and CF_3_ bonds are overlaid onto the CF_2_ bonds’ contribution, which is typical for plasma-based PTFE-like materials. The presence of CF_3_ vibration is confirmed by a peak at 982 cm^−1^, while a small absorption peak at 1433 cm^−1^ is associated with combined asymmetric stretching and rocking deformations of CF_2_ [38]. The FTIR investigation, along with the results obtained from XPS technique, confirms the successful preparation of the CA-GO-TiO_2_/PTFE thin-film composite membranes.

### 3.3. Stability Properties of the CA-GO-TiO_2_ and CA-GO-TiO_2_/PTFE Membranes

#### 3.3.1. Thermal Properties

**Figure 9:**
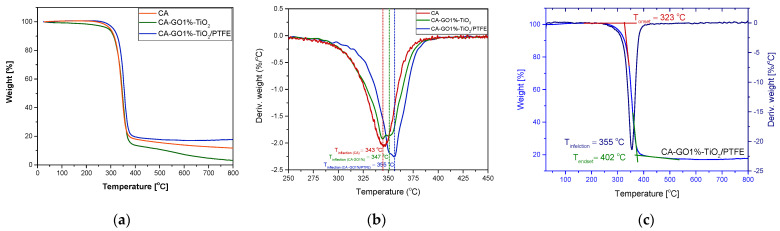
Thermograms of CA-GO1%-TiO_2_/PTFE TFC membranes: TGA (**a**) and DTC (**b**) curves for CA, CA-GO1%-TiO_2_, and CA-GO1%-TiO_2_/PTFE membranes; (**c**) identification of the starting point (T_onset_), the point of the maximum degradation rate (T_inflection_), and the ending point (T_endset_) of the degradation process.

To determine the thermal properties of pristine CA membranes, CA-GO-TiO_2_ composites, and CA-GO-TiO_2_/PTFE thin-film composite membranes, we employed thermogravimetric analysis (TGA) and derivatized thermogravimetric analysis (DTG). This study specifically focused on evaluating how much the incorporation of the graphene oxide (GO) filler and coating with the PTFE-like layer impacted the thermal stability of the newly developed membrane. Figure 9a,b offer insights into the weight loss and derivative weight loss of all the membranes under examination (CA, CA-GO1%, and CA-GO1%/PTFE), while Figure 9c exemplifies, with the CA-GO1%-TiO_2_/PTFE sample, the typical parameters (T_onset_, T_inflection_, T_endset_) used for the characterization of the degradation process. To conclude the findings from the thermal studies, we compiled Table 4, which outlines the initial temperature at which thermal decomposition begins (T_onset_), the temperature at which the maximal degradation rate is observed (T_inflection_), the temperature marking the end of the investigated process (T_endset_), and the quantity of residue remaining.

Upon analyzing the TGA curves, a consistent pattern of thermal degradation becomes apparent in the studied membranes, consisting of a one-step degradation process for all investigated samples. The TGA curves in Figure 9 and the data in Table 4 highlight that incorporating 1% of graphene–titanium dioxide (GO1%-TiO_2_) into the CA membrane led to a delay in the onset temperature, shifting from 308 °C to 312 °C. Further, in the instance of the PTFE-like-coated CA-GO-TiO_2_ membrane, the onset temperature was shifted to an even higher temperature of 323 °C. Analyzing the inflection temperature (T_inflection_) values, we noted a shift from 343 degrees for the initial CA membrane to 347 °C for the CA-GO-TiO_2_ membrane, and to 355 °C for the CA-GO-PTFE membrane. T_endset_ also shifted to higher values from 365 degrees to 367 °C and, respectively, to 372 °C. The increasing trends in the parameters (first, when adding GO-TiO_2_ into the membrane’s composition and further by coating it with PTFE-like material) demonstrate an improved resistance to thermal degradation and greater overall stability of the investigated membranes. By creating a robust and heat-resistant protective barrier, the PTFE-like coating effectively shields the membranes from thermal degradation, ensuring the integrity and performance of the materials, particularly under demanding thermal conditions, such as those appearing during catalytic processes.

#### 3.3.2. Stability of the Membranes with Respect to the Operation in Liquid Phase under UV Irradiation

CA and CA-GO-TiO_2_ membranes dissolve in liquids in the presence of UV light and, therefore, are not suitable for photocatalytic testing [60]. On the contrary, the membranes deposited with PTFE-like thin layers remain intact after many hours. We assign this stability to the protective action and hydrophobic properties of the PTFE-like thin films, which add robustness against the combined effect of UV degradation and dissolution in the liquid.

**Figure 10:**
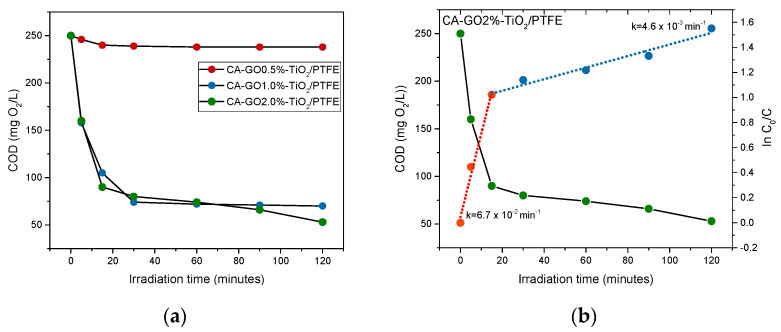
Kinetics of the photocatalytic degradation of the organic content of the Azitrox formulation using the as-obtained CA-GO-TiO_2_/PTFE photocatalytic membranes: (**a**) COD values against time; (**b**) COD values against time and ln(C_0_/C) against time. The graphs illustrate a pseudo-first-order photocatalytic reaction taking place in 2 stages for the CA-GO2%-TiO_2_/PTFE membrane.

The stability of these membranes at working conditions in the photocatalytic reactor is demonstrated by the results obtained from the photocatalytic oxidation tests of the Azitrox formulation. The most straightforward indication of membrane degradation would be the increase in the COD above the initial value given by the Azitrox formulation (250 mg O_2_/L), at least in the first minutes of irradiation. However, such an effect is not noticed. On the contrary, the results (presented in Figure 10a) indicate a decrease in the organic content in the first 15 min of irradiation in all PTFE-like-coated membranes, which is significantly lower for the membranes with 1% GO and 2% GO, respectively. This provides additional evidence in support of the positive role of GO regarding the overall degradation efficiency.

### 3.4. Assessment of Photocatalytic Performances of CA-GO-PTFE Thin-Film Composite Membranes in the Degradation of Azithromycin Formulation

Photo-oxidation experiments were attempted on all samples; however, only PTFE-like-coated membranes were suitable for testing, as all uncoated ones degraded within minutes of being introduced to the reactor. The photocatalytic degradation curves obtained from the three CA-GO-TiO_2_/PTFE membranes, each with a different percentage of TiO_2_-decorated GO, are shown in Figure 10. The data reveal that the decrease in the concentration of organic material is higher during the first 15 min of irradiation. After this period, the concentration only varies slowly. The kinetics of the degradation process are exemplified in Figure 10b for the CA-GO2%-TiO_2_/PTFE membrane.

According to the kinetic behavior of the photocatalytic process, the degradation occurs in two stages with distinct rates. The first stage, which lasts for the first 15 min of irradiation, results in the quick degradation of organic content. In the second stage, the degradation takes place at a slower rate. We emphasize that in this paper, we did not work with pure azithromycin but with an azithromycin formulation, a pharmaceutical product named Azitrox, which, in addition to the active compound azithromycin, contains a lot of excipients (both organic and inorganic) (see Section 2). The presence of organic or inorganic excipients in the Azitrox product are in a permanent competition for the radical species that are formed in the system, and they disturb each other as a result. It seems that the organic excipients from Azitrox formulations, such as sugar, hydroxypropyl cellulose, and starch, play a significant role in the first stage of photocatalytic degradation. These excipients can interact with the free radicals generated in the photocatalytic process by abstracting hydrogens from their molecule, acting as transfer agents of free radicals. As a result, they protect the active component (e.g., azithromycin) from degradation [61]. Once this protection disappears, the active component begins to degrade, and the degradation rate is different. This could be one of the reasons why the organic content of the Azitrox formulation is degraded in two stages with different degradation rates.

Table 5 presents the rate constants of the two degradation stages for each photocatalytic membrane. Specifically, compared to the PTFE-like coated membrane with a lowest concentration (0.5%) of TiO_2_-decorated GO in its composition, the photocatalytic reaction rate constants of the membranes with 1% GO and 2% GO are 14 and 25 times higher, respectively, in the first stage; moreover, they are 9 and 66 times higher, respectively, in the second stage. This result indicates that the degradation rate of organic content in the second stage (azithromycin) increases by one order of magnitude from the PTFE-like-coated membrane containing 0.5%GO-TiO_2_ to the PTFE-like coated membrane containing 1%GO-TiO_2_, and it increases by almost two orders of magnitude relative to that containing 2%GO-TiO_2_.

**Figure 11:**
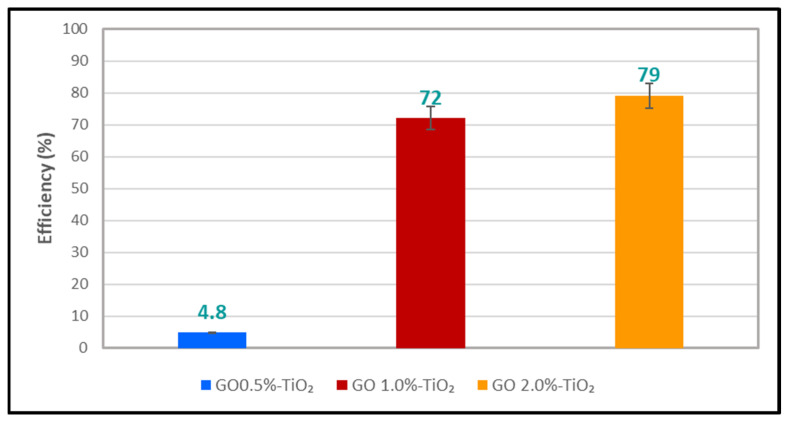
Efficiency of the Azitrox formulation’s degradation via the photocatalytic CA-GO-TiO_2_/PTFE membrane.

In Figure 11, we can see how the efficiency of organic content’s photocatalytic degradation of the Azitrox formulation varies across the three membranes. The membrane with only 0.5%GO-TiO_2_ has a degradation efficiency of less than 5%, which is much lower compared to the other two membranes containing TiO_2_-decorated GO with 1% and 2%). However, the difference in the degradation efficiency between the 1%GO-TiO_2_ and 2%GO-TiO_2_ membranes is only around 7%. This suggests that achieving a higher efficiency of the photocatalytic membrane beyond the maximum percentage obtained (about 80%) does not necessarily require adding more TiO_2_-decorated GO; perhaps, it could depend more on the operating conditions of the photocatalytic reactor.

The results show that the degradation rate of the organic content in both stages increases as the proportion of TiO_2_-decorated graphene oxide in the photocatalytic membrane increases. Therefore, we consider that degradation is driven by the mechanism of TiO_2_-based photocatalysis [2]. In addition, the thin-film composite membranes consist of a combination of GO- TiO_2_ and PTFE-like materials, which can both serve to improve the photocatalytic reaction in two key ways. Firstly, the GO filler amplifies the response of catalytic membranes to UV light [24]. Secondly, at the interface between the PTFE-like material and the TiO_2_ nanoparticles, local electrical fields are generated when the TFC membrane is exposed to UV radiation, thereby providing an additional boost to the degradation reaction [40].

The obtained degradation efficiency of approximately 80% in this study indicates a significantly higher efficiency in the process of degradation compared to the reported degradation rate of 57% for the GO-TiO_2_ composite [24]. Additionally, the results achieved in this study imply that the method utilized here shows promise as a feasible option for the degradation of pollutants. Notably, the potentially higher degradation rate observed in this study compared to the GO-TiO_2_ composite suggests that the method is effective and possibly superior to other ones. No studies have been carried out by other researchers on such complex systems, with the exception of a study also carried out by us, but with a different type of photocatalytic membrane [62] that showed efficiencies up to 70%. Therefore, it is difficult to make a direct comparison of the present photocatalytic degradation process’s efficiency with the one reported for a singular chemical compound, even with azithromycin found alone in an aqueous solution, such as the one that was reported in [63]. On the other hand, the investigated situation better replicates the actual wastewaters [64] originating from our use of Azitrox. This comparison emphasizes the importance of the findings and underscores the method’s potential as a practical solution for mitigating pollution issues.

## 4. Conclusions

We have developed a new type of thin-film composite membrane based on cellulose acetate by incorporating graphene oxide decorated with TiO_2_ in its bulk, and with it being externally coated on both sides with fluorinated (PTFE-like) thin films. The fabrication procedure consists of a liquid processing step producing a homogenous dispersion of GO-TiO_2_ powder in a CA solution, a casting and drying step leading to the generation of CA-GO-TiO_2_ porous substrates via phase inversion, and a vacuum deposition step for coating with PTFE-like films via magnetron sputtering.

We show that the addition of GO-TiO_2_ compounds at a 0.5–2% percentage only slightly influences the porous character-istics of the bulk membrane; nonetheless, at the surface, the chemistry is dominated by carbon–oxygen bonds with a detectable presence of GO. The PTFE-like coating process preserves the porous structure of the bulk but significantly changes surface properties: the surface opening of pores is significantly decreased, and the chemistry becomes dominated by fluorine–carbon bonds.

We demonstrate that the PTFE-like layer provides the membranes with additional chemical and thermal robustness and stability against dissolution in liquids under exposure to UV radiation. Accordingly, photocatalytic degradation studies were possible in the liquid phase under UV irradiation, as performed on Azitrox formulations. Moreover, the PTFE-like films allow for the enhancement of the photocatalytic activity of TiO_2_. The results indicated a two-stage degradation for the Azitrox formulations with different rates, with the first stage being associated with the organic excipients in the formulation and the second stage being associated with the degradation of the active component, namely azithromycin. The azithromycin degradation rate increased by one order of magnitude, changing from the CA-GO0.5%-TiO_2_/PTFE membrane to the CA-GO1%-TiO_2_/PTFE membrane, and it changed by almost two orders of magnitude compared to the CA-GO2%-TiO_2_/PTFE membrane.

## Figures and Tables

**Table 1:** Photocatalytic conditions of experimental tests.

Parameter, Unit	Value
Reactor volume, L	1.5
Recirculation flow rate, L/min	2.0
Volume of AZT working solution, L	2.0
UV lamp	High-pressure mercury lamp; power 120 W
pH of AZT working solution	3
H_2_O_2_/AZT molar ratio	1

**Table 2:** Percentages of pore openings on surface.

Concentration of GO-TiO_2_ in the Sample	Surface Porosity (%)	
	CA-GO-TiO_2_ Samples	CA-GO-TiO_2_/PTFE Samples
0.5%	25.05	15.23
1.0%	24.14	13.24
2.0%	20.86	12.52

**Table 3:** Morphometric parameters registered through micro-CT.

Sample	Structure Thickness (μm)	Closed Porosity (%)	Specific Surface Area (1/μm)	Total Porosity * (%)
CA-GO0.5%-TiO_2_	10.90	0.10	0.31	96
CA-GO1.0%-TiO_2_	11.50	0.25	0.28	89
CA-GO2.0%-TiO_2_	12.70	0.24	0.27	90
**Averaged values** **CA-GO-TiO_2_**	**11.7**	**0.19**	**0.28**	**91.6**
CA-GO0.5%-TiO_2_/PTFE	17.20	0.14	0.19	88
CA-GO1.0%-TiO_2_/PTFE	11.30	0.29	0.31	84
CA-GO2.0%-TiO_2_/PTFE	10.90	0.39	0.30	91
**Averaged values** **CA-GO-TiO_2_/PTFE**	**13.13**	**0.27**	**0.26**	**87.6**

* Computed from μCT data for the entire volume of the sample.

**Table 4:** Thermogravimetric characteristics for CA, CA-GO1%-TiO_2_, and CA-GO1%-TiO_2_/PTFE membranes.

Sample/ Significant T (°C)	CA	CA-GO1%-TiO_2_	CA-GO1%-TiO_2_/PTFE
T_onset_	308	312	323
T_inflection_	343	347	355
T_endset_	365	367	402

**Table 5:** Pseudo-first-order rate constants for the photocatalytic degradation of the organic content corresponding to the three variants of photocatalytic membranes; r^2^ denominates the residual sum of squares for the 2nd stage of each photocatalytic process.

Pseudo-First-Order Rate Constants (k, min^−1^)			
Sample Codes	Degradation Stage		
	1st Stage	2nd Stage	Residual Sum of Squares for the 2nd Stage (r^2^)
CA-GO0.5%-TiO_2_/PTFE	2.7 × 10^−3^	7.0 × 10^−5^	1.7 ×10^−5^
CA-GO1%-TiO_2_/PTFE	3.8 × 10^−2^	6.0 × 10^−4^	6.3 ×10^−2^
CA-GO2%-TiO_2_/PTFE	6.7 × 10^−2^	4.6 × 10^−3^	5.4 × 10^−3^

## Data Availability

The original contributions presented in this study are included in this article/; further inquiries can be directed to the corresponding authors.
